# Psychometric Evaluation of the Multidimensional Scale of Perceived Social Support Among Early Adolescents in Darjeeling, India

**DOI:** 10.3390/ejihpe15120251

**Published:** 2025-12-09

**Authors:** Megan Cherewick, Michael Matergia, Choden Dukpa, Dikcha Mukhia, Rinzi Lama, Roshan P. Rai, Priscilla Giri

**Affiliations:** 1 Department of Community and Behavioral Health, Colorado School of Public Health, University of Colorado Anschutz Medical Campus, 3001 E 17th Pl, Aurora, CO 80045, USA; 2 Broadleaf Health and Education Alliance, Denver, CO 80202, USA; michael.matergia@broadleafhea.org; 3 Darjeeling Ladenla Road Prerna, Darjeeling 734101, West Bengal, India; 4 Department of Anthropology, University of North Bengal, Siliguri 734014, West Bengal, India; rinzi.lama@nbu.ac.in

**Keywords:** social support, adolescence, psychometric analysis, LMIC

## Abstract

Background. The Multidimensional Scale of Perceived Social Support (MSPSS) is widely used, although it has not been validated among early adolescents in Darjeeling, India. The aims of the study were to validate the psychometric properties of the MSPSS, and to test for measurement invariance by gender. Methods. Survey data was collected from 274 early adolescents ages 10–14 living in Darjeeling, India. Confirmatory factor analysis (CFA) evaluated 1-, 2-, and 3-factor models. Reliability (Cronbach’s α, McDonald’s ω), convergent (peer problems), and concurrent validity (prosocial behavior) were assessed. Measurement invariance by gender was tested using multi-group CFA. Results. The three-factor model of the MSPSS (Family, Friends, Significant Other) fit these data well (X^2^[49] = 69.3, *p* = 0.030; CFI = 0.98; RMSEA = 0.039; SRMR = 0.036). Measures of reliability, concurrent, and convergent validity were good with MSPSS scores correlated positively with prosocial behavior and negatively with peer problems (|r| = 0.30–0.45, *p* ≤ 0.001). Configural invariance by gender was not supported, indicating differences in item-level loadings. Limitations. The MSPSS is a self-report measure, and social desirability bias is a potential limitation. Conclusion. The MSPSS demonstrates good reliability and validity among early adolescents in Darjeeling, India. Given non-invariance by gender, subscale comparisons across boys and girls should be interpreted with caution.

## 1. Introduction

Early adolescence is a developmental period marked by rapid changes in neural systems that affect motivation, emotional regulation, and social orientation (Blakemore & Mills, 2014; Braams et al., 2015; Cherewick et al., 2024; Crone & Dahl, 2012; Davidson & McEwen, 2012; Hankin et al., 1998; Kessler et al., 2005; Lam et al., 2014; McCarthy et al., 2016; Orben et al., 2020; Pfeifer & Berkman, 2018; Shulman et al., 2016; Steinberg, 2008; Twenge & Nolen-Hoeksema, 2002). This social orientation is not merely descriptive, but it reflects neurobiological changes in reward and threat systems that drive early adolescent development and behavioral patterns (Bloom et al., 2025; Dahl et al., 2018; Flament et al., 2007; McGorry & Mei, 2018; McGorry et al., 2018; Tottenham & Galván, 2016). Social support has been widely studied as a protective factor for mental health and wellbeing, and conversely, lack of social support has been shown to predict poor mental health outcomes in adolescents (Chu et al., 2010; M. Guan & So, 2016; Kleinberg et al., 2013; Marshall et al., 2014; Miller et al., 2015; Ronen et al., 2016; Sharaf et al., 2009).

Foundational theory delineates two complementary pathways by which social support affects health: a main-effect pathway (benefits at baseline) and a stress-buffering pathway (attenuation of stress effects) (Cohen & Wills, 1985). Classic frameworks distinguish structural features of networks from functional provisions, while contemporary theory (Cohen & Wills, 1985) examines the social-cognitive processes specified by functional forms (e.g., emotional, informational, and instrumental) and how they influence appraisal, coping, and action (Beckes & Coan, 2011; Cohen & Wills, 1985; Cohen & Syme, 1985; Lakey & Cohen, 2000; Ryan & Deci, 2000; Schwarzer & Knoll, 2007; Thoits, 2011).

Main effect models propose that having supportive relationships yields benefits at all times by fostering positive affect, adaptive norms, and healthful routines (Cohen & Wills, 1985). The stress-buffering model posits that support primarily protects individuals during times of adversity, so that when stressors arise, available and credible support reduces threat appraisal, improves coping, and attenuates physiological reactivity (Cohen & McKay, 2020; Cohen & Wills, 1985).

Social-cognitive oriented theory clarifies how these pathways operate by specifying functional forms of support (emotional, informational, and instrumental) and the processes through which they influence health behavior and adaptation (Schwarzer & Knoll, 2007). Emotional support shapes appraisal and emotion regulation; informational support promotes problem solving and self-management; instrumental support removes practical barriers to action (Schwarzer & Knoll, 2007). These processes map closely onto early adolescent tasks (learning to regulate affect, handle peer challenges, and navigate school demands), and they underscore that the source of the support may be important for mechanistic strength (e.g., parents for instrumental support; friends for emotion regulation and belonging) (Ciarrochi et al., 2017).

Theory further distinguishes perceived from received support. The perception that support will be provided if needed often predicts outcomes more strongly than instances of help actually being provided, because it is proximal to appraisal, expectancy, and regulation (Lakey & Cohen, 2000). Self Determination Theory specifies relatedness as a basic psychological need; contexts that satisfy relatedness promote adaptive motivation and prosocial engagement, whereas lower relatedness elevates risk for distress (Ryan & Deci, 2000). Social Baseline Theory complements this account, proposing that humans expect close relatives to help regulate threat and conserve effort; meanwhile, social proximity down-regulates physiological arousal and attenuates stress responses (Beckes & Coan, 2011; Coan et al., 2014). In early adolescence, when threat appraisal becomes labile and social evaluation carriers higher weight, differences in perceived support from family, friends, and significant others is a theoretically proximal driver of stress appraisal and regulation, which is why it matters who is the source of the perceived support (Lakey & Cohen, 2000).

Because of the importance of social support in predicting mental health and wellbeing, more research has begun to evaluate sources of social support (Bernaras et al., 2019; Cohen & McKay, 2020; Morriss & Morriss, 2000; van den Berg et al., 2013; Winefield et al., 1992). Consistent with source-specific theories, adolescent studies across contexts and across settings, including low-and middle-income countries (LMICs), typically find distinct family, friends, and significant other dimensions of perceived social support (Alshammari et al., 2021; Canty-Mitchell & Zimet, 2000; Edwards, 2004; Glozah, 2013; M. Guan & So, 2016; N. C. Guan et al., 2015; Robu et al., 2020). Family support is often tied to routine scaffolding and protection; peer support is closely related to emotion regulation and belonging; and significant other support captures confidant-type bonds in early adolescence. Deficits in perceived family support have predicted depressive symptoms and suicidal ideation/attempts, whereas higher family support has been shown to be negatively correlated with severity of depressive symptoms (Sharaf et al., 2009). Developmentally, by mid-to-late adolescence, friends increasingly rival or exceed parents as central support figures, and romantic partners also rise in importance, creating variability in how support sources are perceived during the transition from early to mid-adolescence (Furman & Buhrmester, 1992; Nelson et al., 2005). Longitudinal evidence from Australia found peer support predicted mental wellbeing even when family and significant other social support levels were low (Ciarrochi et al., 2017).

The Multidimensional Scale of Perceived Social Support (MSPSS) operationalizes perceived social support from three sources: (1) family; (2) friends, and (3) significant others (Zimet et al., 1988). In many high-income countries (HIC), the MSPSS has shown to be best represented by the original 3-factor analytic solution with subscales demonstrating satisfactory reliability in adolescent populations. However, the MSPSS is not invariant across all youth contexts. Several LMIC and Asian adolescent studies report both three-factor and two-factor solutions in which friends and significant other load together, or show item-level deviations, often without loss of reliability (Akhtar et al., 2010; Aloba et al., 2019; Cheng & Chan, 2004; Chou, 2000; Edwards, 2004; Ramaswamy et al., 2009; Wilson et al., 2017). These departures are theoretically interpretable: in early adolescence, “significant other” may be construed as a best friend or trusted adult rather than a romantic partner. Extended family caregiving can broaden the meaning of “family”, and school anchored relationships (e.g., teachers/near-peers) can function as close confidants (Wilson et al., 2017).

Building on this theoretical and empirical groundwork, we conduct a context-specific psychometric evaluation of the MSPSS among early adolescents in Darjeeling, India. Darjeeling, India is a minoritized region of India characterized by economic migration, political instability, and poverty. In this setting, non-traditional family and changing family structures are common. For these reasons, the source of social support from family, friends, or significant others may be different than in other adolescent samples in different contexts (Canty-Mitchell & Zimet, 2000). Extended family caretakers are common, and thus significant others may be indistinguishable from family, supporting a two-factor structure. Alternatively, extended family may be viewed as an important source of support, separate from family.

### Objectives

In this study, we conceptualize social support as a protective factor whose effects can appear as both main effects and stress-buffering effects. We operationalize social support as perceived availability of support (MSPSS), which is proximally linked to appraisal and regulation and often predicts outcomes more strongly than actualized help (Lakey & Cohen, 2000). We (1) compare one, two, and three factor structures of the MSPSS and estimate reliability; (2) assess convergent (peer problems) and concurrent (prosocial behavior) validity; and (3) explore measurement invariance by gender using multi-group CFA, given theory-driven reasons to anticipate subgroup differences in how social support sources are perceived.

## 2. Methods

### 2.1. Setting

The study sample included four low-cost private (LCP) schools representing urban, peri-urban, and rural communities in the Darjeeling Himalayas, a district of the state of West Bengal in India. Darjeeling is geographically and ethnically distinct, composed of Indian citizens of Nepali descent, other ethnic groups, as well as economic migrants from adjoining states and districts. Socioeconomic conditions vary but are generally constrained, with most adults earning less than $2.76 USD per day (Gorkhaland Territorial Administration, 2018; Rai, 2017). The local economy is anchored in tea production, tourism, small-scale agriculture, education, and military service, and a large share of residents work in the informal sector. The economy of Darjeeling is significantly dependent on remittance accrued from seasonal and long-term migration of residents to other states within India and abroad. Unlike many districts in India, women’s participation in both formal and informal economic activities is central to Darjeeling’s economy.

In urban Darjeeling, LCP schools enroll approximately 30–50% of all primary school students in India, educate a large proportion of lower-income adolescents, and serve families with diverse migration histories (Gorkhaland Territorial Administration, 2018; Matergia et al., 2019; Rai, 2017). Partnering with LCP schools through our local NGO (Darjeeling Ladenla Road Prerna, DLRP) enabled feasible, culturally supported implementation (e.g., language, scheduling, parental permissions) for a context-specific validation.

### 2.2. Participants

Four schools were recruited to participate in the study based on positive working relationships with the local non-governmental organization and implementation team, Darjeeling Ladenla Road Prerna (DLRP). Together with school leaders, DLRP identified classrooms with adolescents who met eligibility criteria, and classrooms were selected using a random number generation. Within each class, all eligible participants were invited to participate. Eligibility criteria included: (1) age 10–14 upon enrollment, (2) lives in Darjeeling, India, (3) attends 1 of 4 low-cost private schools, (4) caregiver written consent, and (5) participant verbal assent. There were no exclusion criteria.

Informed written consent was obtained from caregivers of all adolescents and verbal assent obtained from all adolescent participants prior to data collection activities. Participants were given a description of the study, and were told that their participation was voluntary and that they were allowed to withdraw from the study at any time. Participants had the opportunity to ask questions and provide an affirmative verbal indication of their assent to participate in the study. Participants completed 45-min surveys administered after school on school grounds in a private space. Research assistants read each question aloud in Nepali while participants recorded their responses on a corresponding written survey on paper which were provided in Nepali or English (if preferred). After completing the survey, the research assistants recorded participant responses in the secure web application, the Research Electronic Data Capture application (REDCap) hosted by the University of Colorado.

### 2.3. Measures

#### 2.3.1. The Multidimensional Scale of Perceived Social Support (MSPSS)

The MSPSS self-report scale was developed by Zimet et al. (1988) to assess three dimensions of social support through twelve items (Zimet et al., 1988). The family support dimension consists of items 3, 4, 8, and 11. The friends dimensions consists of items 6, 7, 9, and 12. The significant others dimension consists of items 1, 2, 5, and 10. Participant responses were recorded on a 7-point Likert scale ranging from 1 = strongly disagree to 7 = strongly agree, with higher scores indicating greater perceived support. Examples of items for the friends subscale include: “I have friends with whom I can share my joys and sorrows,” and “I can talk about my problems with my friends.” Examples of items for the family subscale include: “My family really tries to help me,” and “I get the emotional help and support I need from my family.” Examples of items for the significant others subscale include: “There is a special person in my life who cares about my feelings,” and “There is a special person who is around when I am in need.” Total scores range from 12 to 84. Cronbach’s alpha in this sample for the full scale was 0.85.

#### 2.3.2. Strengths and Difficulties Questionnaire (SDQ)

The SDQ is a 25-item questionnaire to assess mental health problems in children ages 11–16 (Goodman, 1997). The questionnaire assesses five dimensions including conduct problems, hyperactivity-inattention, emotional symptoms, peer problems and prosocial behavior. In this study, the peer problems subscale was used to examine convergent validity, and the prosocial subscale was used to assess concurrent validity. Examples of peer problem subscale items include “I would rather be alone than with people my age” and “Other children or young people pick on me or bully me.” Example items for the prosocial subscale include: “I often offer to help others” and “I try to be nice to other people. I care about their feelings.” Response categories include three answer choices (0 = not true; 1 = somewhat true; 2 = certainly true). The Cronbach’s alpha for the SDQ in this sample was 0.73. Although the SDQ self-report is most commonly used from ages 11–17, psychometric work shows the youth self-report is usable in ages 8–13 years, supporting inclusion of 10-year olds when comprehension is supported by administration procedures (e.g., items read aloud, language choice) (Muris et al., 2004).

We selected the SDQ to provide construct validity anchors for the MSPSS because its peer problems subscale captures difficulties in peer relations that should relate inversely to perceived social support (convergent validity), while its prosocial subscale indexes helping/considerate behavior that should relate positively to perceived social support (concurrent validity). The SDQ’s brevity, cross-cultural use (including in India), language availability, and suitability for RA (read-aloud administration) made it feasible in the after-school setting of this study.

#### 2.3.3. Additional Measures

Additional demographic variables measured include sex, age, school, and class level.

### 2.4. Data Analysis

Data analysis was performed using Stata version 14. There was minimal missing data in the sample for measured variables (1.4%), and analysis compared missing and non-missing data for age and sex and determined that missing data was not statistically related to age and sex. Therefore, analyses proceeded with complete cases (n = 274). The sample size was deemed adequate following: (1) conventional requirements for confirmatory factor analysis of 5–10 study subjects for each item analyzed, and meeting high subject to item ratios of 20:1 (Costello & Osborne, 2019; Tinsley & Tinsley, 1987); and (2) methodological simulations indicating that the required sample size in CFA depends on model parameters (e.g., indicators per factor, loading magnitude, estimator); with four indicators per factor and moderate loadings falling in the range of N = 200–300 or lower (Kyriazos, 2018; Wolf et al., 2013). Descriptive statistics included means and standard deviations of measured variables presented by gender. Our analytical approach to factor analysis applied confirmatory factor analysis (CFA) because the purpose of the present study was to test if these data were best fit with a one, two, or three-factor analytical model found in prior studies in LMIC and following recommended methodological analyses for CFA (Hurley et al., 1997; Orçan, 2018).

Confirmatory factor analysis (CFA) was performed using structural equation modeling and maximum likelihood estimation to evaluate whether previously reported factor structures of the MSPSS fit these data adequately. A one, two, and three factor model were fit and evaluated using conventional fit criteria. Fit indices included: comparative fit index (CFI) greater than 0.90, Tucker Lewis index (TLI) > 0.90, the root mean square error of approximation (RMSEA) less than 0.06, and the standardized root mean square residual (SRMR) less than 0.08 (Bentler, 1990; Hu & Bentler, 1999; Steiger, 1980; Tucker & Lewis, 1973). Next, correlational analyses assessed criterion validity of the model with the best fit. Concurrent validity was examined for significant correlation with the prosocial subscale of the SDQ. Convergent validity was assessed through correlational analysis with the peer-problems subscale of SDQ. Internal reliability was assessed using Cronbach’s alpha (α) and McDonald’s omega (Ω) (Nunnally & Bernstein, 1978).

Measurement invariance by gender was explored via multi-group CFA in three steps: configural (same factor structure), metric (factor loadings constrained equal), and scalar (factor loadings and intercepts constrained equal). We compared each constrained model to the less constrained model using changes in fit indices. Following recommended guidelines, recommended cutoffs supporting measurement invariance include: ΔCFI < 0.01, ΔTLI < 0.01, ΔRMSEA < 0.015, and ΔSRMR < 0.03 in comparison to the baseline configural model (Chen, 2007; Cheung & Rensvold, 2002). The exploratory test for configural invariance was not supported; therefore, more restrictive models were not pursued.

### 2.5. Ethics Approval

This study was approved by the Colorado Multiple Institutional Review Board (COMIRB), Protocol approval Number: 23-1421, and the local ethics review board, St. Joseph’s College North Pointe Ethics Committee (SJC-NP-EC).

## 3. Results

### 3.1. Descriptive Statistics

The sample comprised 274 participants, 135 female and 139 male early adolescents ages 10–14. Counts, percentages, means, and standard deviations comparing girls and the total sample are presented in Table 1. Chi-squared tests were completed to assess for differences by gender in age, class, and degree of urbanization. *p*-values indicate there were no significant differences by gender.

### 3.2. Factor Analysis

**Figure 1:**
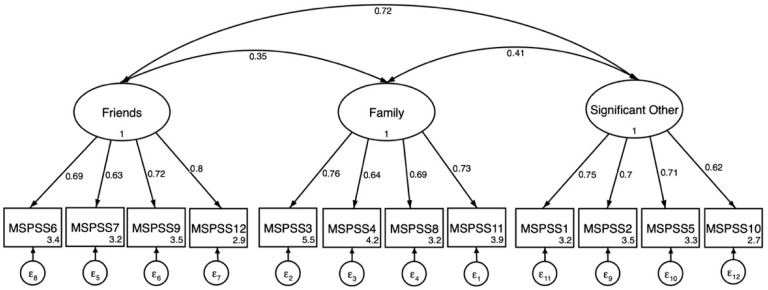
Three-factor confirmatory factor analysis of the MSPSS (N = 274). All factor loadings on latent factors are significant at the *p* ≤ 0.001 level. Values inside item boxes represent intercepts.

Confirmatory factor analysis was completed using the analytical sample (N = 274). Confirmatory factor analysis indicated that a one-factor model did not fit these data adequately (CFI = 0.69; TLI = 0.62; SRMR = 0.116; RMSEA = 0.155). Examination of fit indices also indicated that a two-factor model did not fit these data well (CFI = 0.89; TLI = 0.87; SRMR = 0.060; RMSEA= 0.090). A three-factor solution indicated excellent fit; *X*^2^ = 69.3 (49), *p*-value = 0.030; CFI = 0.98; TLI = 0.98; SRMR = 0.036; and RMSEA = 0.039. The standardized factor loadings and item communality (R^2^) on friends, family, and significant other subscales is presented in Table 2. A CFA path diagram representing item loadings are depicted in Figure 1. For girls, with a three-factor solution, standardized factor loadings (λ) were moderate to strong across items; for boys items also loaded moderately to strongly on their intended factors in the three-factor model.

### 3.3. Configural and Measurement Invariance

**Figure 2:**
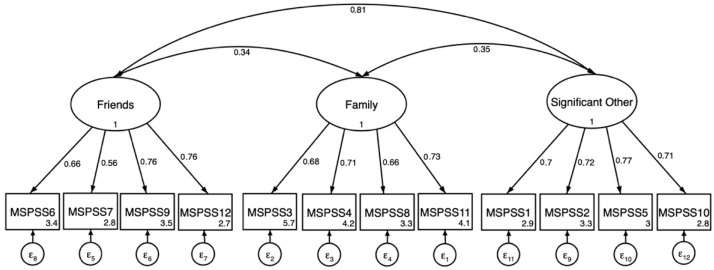
Three-factor confirmatory factor analysis of the MSPSS (N = 139) in males. All factor loadings on latent factors significant at the *p* < 0.001 level. Values inside item boxes represent intercepts.

**Figure 3:**
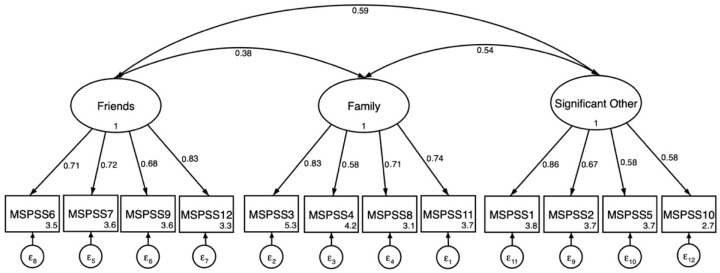
Three-factor confirmatory factor analysis of the MSPSS (N = 135) in females. All factor loadings on latent factors significant at the *p* < 0.001 level. Values inside item boxes represent intercepts.

Results from fitting the 3-factor model separately for boys and girls are presented in Table 3. Compared to the total sample 3-factor results, the 3-factor confirmatory factor analysis results for males showed a slight but acceptable decrease in model fit indices (CFI = 0.98; TLI = 0.97; SRMR = 0.060; RMSEA = 0.040). However, for females, the model fit indices reduced more substantially (CFI = 0.95; TLI = 0.94; SRMR = 0.060; RMSEA = 0.067). CFA path figures are presented separately for males and females in Figure 2 and Figure 3 respectively. Results from configural invariance tests indicated that this level of constraint was not supported according to predetermined cutoff results of configural invariance tests (Chen, 2007). Because configural invariance tests are the least constrained model, the test showed that even this baseline model did not fit well across groups. Accordingly, tests for metric and scalar invariance did not proceed. After completion of the configural invariance test, modification indices were examined to explore potential sources of configural invariance. Examination of CFA results and modification indices (MI) indicated differences in covariance and factor loading paths.

### 3.4. Internal Consistency

Reliability was assessed by evaluating Cronbach’s alpha and McDonald’s omega (Hayes & Coutts, 2020; Tavakol & Dennick, 2011). The reliability for the total scale was excellent (α = 0.85; Ω = 0.84). The friends subscale demonstrated good reliability (α = 0.80; Ω = 0.80). The family subscale demonstrated good reliability (α = 0.79; Ω = 0.80). The significant others subscale demonstrated good reliability (α = 0.79; Ω = 0.79).

### 3.5. Concurrent and Convergent Validity

Concurrent validity was established using the prosocial subscale of the Strengths and Difficulties Questionnaire (SDQ). It was expected that the relationship between the MSPSS subscales and prosocial behavior would be positive and the relationship between the MSPSS subscales and peer problems would be negative. Correlations between subscales of the MSPSS and the total MSPSS scale with prosocial behavior are listed in Table 4. Convergent validity was tested by assessing significant correlations between the MSPSS subscales and peer problems subscale of the SDQ. All correlational analyses were highly significant at the *p* ≤ 0.001 level demonstrating excellent concurrent and convergent validity.

## 4. Discussion

Perceived social support is a well-established protective factor for adolescent mental health, operating through both main-effect and stress-buffering pathways and delivered via functionally distinct forms of help (emotional, informational, instrumental). Grounding our analysis in source-specific assessment matters in early adolescence: providers differ in what they can offer and in developmental salience, with peers becoming increasingly central and “significant others” often functioning as close confidants (rather than romantic partners) at these ages. The primary objective of this study was to evaluate the psychometric properties of the MSPSS in a sample of early adolescents living in Darjeeling, India.

Results indicate that the MSPSS is a reliable and valid instrument for measuring social support in the context of Darjeeling. Confirmatory factor analyses (CFA) supported the original three-factor structure (family, friends, significant other) of the MSPSS in early adolescents from Darjeeling, India, with good model fit and internal consistency across subscales. Analysis of reliability (internal consistency) was strong using both Cronbach’s alpha and McDonald’s omega. While some have argued that McDonald’s omega provides superior estimates of internal consistency in comparison to Cronbach’s alpha, in our sample, both measures produced similar estimates. Concurrent validity was supported by positive associations with prosocial behaviors, and convergent validity by inverse associations with peer problems with correlational coefficients ranging from 0.30–0.45. In our study, the direction, strength, and significance of correlations between the MSPSS and other measures confirmed excellent concurrent and convergent validity.

Our exploratory test of measurement-invariance by gender did not support configural invariance, indicating that the pattern of item-factor relations differs for boys and girls in this context. Given per group sample sizes, these analyses were underpowered to detect small cross-group differences; accordingly, we interpret the result as a caution against assuming cross-gender equivalence, rather than as a definitive map of which parameters diverge. Substantively, this aligns with developmental theory that the rank order and meaning of support providers shift with age and differ by sex during adolescence, altering how items referencing friends, family, or a significant other are construed. Methodologically, latent means or pooled factor scores should not be compared across gender until at least partial invariance is established. Future research in this setting should include larger, balanced samples to test metric/scalar (partial) invariance, consider DIF-sensitive approaches, and use cognitive interviewing to probe items whose wordings may vary in meaning by gender.

Item level patterns illustrate these points. In our study, females held higher factor loadings for social support from friends in comparison to males. This finding is similar to studies indicating that adolescent females reported greater social support from friends (Bruwer et al., 2008; Canty-Mitchell & Zimet, 2000; Gecková et al., 2003; Klineberg et al., 2006). These finding are unlike other studies completed in a LMIC that supported measurement invariance by gender. A study in Nigerian adolescents confirmed measurement invariance by gender was completed in an analytic sample with a slightly higher age range (mean age: 15 years). Similarly, a study completed with Indonesian adolescents (mean age: 15 years) found measurement invariance of the MSPSS by gender (Laksmita et al., 2020). It is plausible that at younger ages, males and females may understand item level questions differently, but approach equivalence in their understanding as they develop (Aloba et al., 2019). Other studies have found that males had greater levels of “school-related” social support compared to females because of sociocultural expectations of females being closer to family and males having more freedom outside the home (Buchanan, 2014). In our study, two significant other items (MSPSS 1, MSPSS 2) showed near-threshold cross-loadings on friends (λ = 0.31 and 0.34), below but close to our a priori threshold (λ >= 0.35). This is consistent with the developmental and cultural context of Darjeeling in which a “significant other” may be a best friend or kin-like peer, thereby softening the boundary between friends and significant other in early adolescence. With overall model fit supported by the three-factor solution, these cross-loadings suggest caution in assuming strong discriminant validity between these two sources in this setting and highlight the need for qualitative work and/or psychometric testing in a larger sample.

In the present study, for males the lowest factor loading was 0.56 on the friends subscale; item 7: “I can count on my friends when things go wrong”). A study with Indonesian adolescent disaster survivors found also found this item to be lowest (for both genders) and the authors suggest that this may have been caused by misinterpretation of what “counting” on friends means when having a problem. While it is possible this question could be misinterpreted in our sample, we suggest that the nature of the problem itself may be a reason for not endorsing this item. For example, if a problem is financially related, friends may not be seen as source support. The factor loading for this item was higher for females (0.72) and may reflect the differences in the types of problems girls identify when answering this question in comparison to boys. More research is needed to contextualize how perceived social support may be related to the type of challenge or stressor experienced.

For girls, the lowest factor loading was shared among three items (0.58), i.e. the family subscale, item 4: “I get the emotional help and support I need from my family”, and the significant others subscale, item 5: “I have a special person who is a real source of comfort to me” and item 10: “There is a special person in my life who cares about my feelings”. While these three items represent two subscales, the items represent emotional support/comfort. Girls in this context may receive more emotional support from friends than family and significant others in comparison to boys who had factor loadings higher than 0.70 on these items. Again, the type of social support received may be important to understand better how adolescents perceive social support in response to needs and challenges.

Interventions to facilitate high-quality relationships should examine whether these sources of support are used to meet a variety of support needs such as emotional, social, academic, and financial needs. Research can help support precision in the content and delivery of interventions. For example, if girls are lacking in emotional support from family and significant others, this may reflect a need for interventions that focus on open and positive communication with trusted adults. If males feel they lack support from friends, more research is needed to understand if that support is situationally grounded (e.g., need for financial support). Further, given the high positive correlation between the MSPSS and prosocial behavior and inverse correlation with peer problems, intervening during early adolescence may be a critical opportunity to bolster the protective and promotive effects of social support before behavioral patterns emerge that increase risk for mental health and wellbeing.

Psychometric validation of the MSPSS is not only useful for analyses seeking to assess independent relationships with the mental health outcomes of each subscale, but the MSPSS can also be used to assess the effects of interaction between subscales. Further, these effects should evaluate both protective (stress buffering) and promotive effects on mental health. In other LMIC, MSPSS scores have been inversely related to measures of mental distress and depression, anxiety, higher exposure to community violence and trauma, and other mental health conditions (Akhtar et al., 2010; Bruwer et al., 2008; Poudel et al., 2020). Studies also show a positive correlation between social support from friends and resilience (Bruwer et al., 2008).

These findings map onto actionable program implications in India’s policy environment. The Rashtriya Kishor Swasthya Karyakram (RKSK) program broadened adolescent health beyond sexual/reproductive health to include mental health, nutrition, injuries/violence, NCD risk, and substance misuse delivered through Adolescent Health Clinics and community/school platforms, including the Saathiya peer-educator program. RKSK now also interfaces with the School Health and Wellness Programme in India (Jain et al., 2019; Kansara et al., 2018; Nandan, 2010; Pandey et al., 2020). In this delivery landscape, source-specific MSPSS subscales are programmatically actionable: lower support from friends can flag the need for peer-focused Saathiya sessions or school-based group activities; lower family support can guide caregiver outreach or family counselling; and patterns in significant other items, often reflecting confidants at these ages, can inform referral pathways and mentorship supports. Validating the MSPSS locally helps ensure that these signals are interpreted correctly for targeting, counselling, and referral in Darjeeling.

Interventions to strengthen high-quality social relationships should also consider function–source matching (e.g., emotional vs. informational vs. instrumental needs) and developmental timing. For example, if girls report lower emotional support from family/significant others, programming can target open, supportive communication with trusted adults. If boys perceive lower friend support, formative work should examine whether this reflects situational constraints (e.g., practical/financial stressors where peers are not expected to help) and address those gaps through structured peer-support or linkage to material supports. Given the positive correlation between perceived support and prosocial behavior and the inverse association with peer problems, intervening in early adolescence may leverage a window to reinforce protective social processes before maladaptive patterns consolidate.

### Limitations

This study included several limitations. First, the cross-sectional design precludes temporal inference. Second, the MSPSS is a self-report measure, and it is possible participants were biased by social desirability factors. Additional measures by caregivers or teachers could further support validation of the dimensional structure of the measure. Third, due to failure to support measurement invariance by gender, it is recommended that additional research seek to understand differences in item-level understanding by gender. Fourth, our sampling frame was limited to LCP schools which may limit generalizability to government or higher-fee private schools. Finally, broader clinical measures for validation (e.g., internalizing symptoms) were not included, however future work should incorporate additional validation measures and test invariance across school types and languages.

## 5. Conclusions

These findings contribute to a growing body of research underscoring the important relationship between dimensions of social support and mental health and wellbeing outcomes. In a minoritized, lower-income region of the Indian Himalayas, adolescents’ perceptions of social support are structured in ways that matter for both measurement and intervention. By situating MSPSS evaluation in theory, testing competing structures, and linking source-specific scores to feasible delivery channels, this study advances a context-appropriate foundation for using perceived support as a practical signal for adolescent mental health programming in Darjeeling.

## Figures and Tables

**Table 1:** Descriptive statistics of the analytical sample (N = 274).

	Females N = 135	Males N = 139	Total N = 274	*p*-Value
Age				0.447
10	11	13	24	
11	25	30	55	
12	29	30	59	
13	31	29	60	
14	39	37	76	
Mean (SD)	12.5 (1.3)	12.3 (1.3)	12.4 (1.3)	
Class				0.123
5	17	24	41	
6	28	28	56	
7	23	29	52	
8	32	34	66	
9	25	19	44	
10	9	5	14	
Location				0.335
Urban	15	4	19	
Peri-urban	22	32	57	
Rural	98	100	198	

**Table 2:** Confirmatory factor analysis (CFA) standardized loadings (λ) and communality for the Multidimensional Scale of Perceived Social Support (MSPSS), by gender.

	Factors			
MSPSS Item	Friends	Family	Significant Other	Communality
6	**0.61**	0.15	0.22	0.45
7	**0.55**	0.11	0.23	0.37
9	**0.70**	0.16	0.19	0.55
12	**0.74**	0.06	0.31	0.65
3	0.04	**0.78**	0.09	0.62
4	0.08	**0.60**	0.20	0.41
8	0.17	**0.63**	0.16	0.45
11	0.16	**0.73**	0.05	0.57
1	0.31	0.20	**0.62**	0.52
2	0.34	0.17	**0.55**	0.46
5	0.26	0.07	**0.72**	0.58
10	0.23	0.13	**0.59**	0.42

Note: Factor loadings (λ) > 0.35 are in bold; communality (item R^2^) = 1 − θ where θ is the standardized residual variance for each item.

**Table 3:** Measurement invariance tests by gender (3-factor MSPSS).

	*X*^2^ (df)	*p*-Value	CFI	ΔCFI	TLI	ΔTLI	SRMR	ΔSRMR	RMSEA	ΔRMSEA
Total	69.3(49)	0.030	0.98	REF	0.98	REF	0.036	REF	0.039	REF
Males	62.8 (49)	0.106	0.98	0.00	0.97	−0.01	0.046	0.010	0.043	0.004
Females	76.7 (49)	0.005	0.95	−0.03	0.94	−0.04	0.060	0.024	0.067	0.028
Configural	174.2 (116)	0.000	0.95	−0.03	0.94	−0.04	0.073	0.047	0.061	0.022

Note. CFI: Comparative Fit Index; TLI: Tucker Lewis Index; SRMR: Standardized Root Mean Square Residual; RMSEA: Root Mean Square Error of Approximation.

**Table 4:** Pearson correlation coefficients of MSPSS dimensions and measures to evaluate concurrent and convergent validity.

	Concurrent Validity	Convergent Validity
*MSPSS*	Prosocial Behavior	Peer Problems
Friends	0.30 ***	−0.38 ***
Family	0.35 ***	−0.37 ***
Significant Other	0.31 ***	−0.30 ***
Total	0.41 ***	−0.45 ***

Note. *** *p* ≤ 0.001.

## Data Availability

The data presented in this study are available on request from the corresponding author to ensure data safety.
